# Feasibility of PRIME: A Cognitive Neuroscience-Informed Mobile App Intervention to Enhance Motivated Behavior and Improve Quality of Life in Recent Onset Schizophrenia

**DOI:** 10.2196/resprot.5450

**Published:** 2016-04-28

**Authors:** Danielle Schlosser, Timothy Campellone, Daniel Kim, Brandy Truong, Silvia Vergani, Charlie Ward, Sophia Vinogradov

**Affiliations:** ^1^ University of California, San Francisco Department of Psychiatry San Francisco, CA United States; ^2^ University of California, Berekeley Berkeley, CA United States; ^3^ IDEO Palo Alto, CA United States; ^4^ San Francisco Veterans Affairs Medical Center San Francisco, CA United States

**Keywords:** schizophrenia, mobile app, smartphone, motivation, technology-based intervention, social networking, coaching, negative symptoms, quality of life

## Abstract

**Background:**

Despite improvements in treating psychosis, schizophrenia remains a chronic and debilitating disorder that affects approximately 1% of the US population and costs society more than depression, dementia, and other medical illnesses across most of the lifespan. Improving functioning early in the course of illness could have significant implications for long-term outcome of individuals with schizophrenia. Yet, current gold-standard treatments do not lead to clinically meaningful improvements in outcome, partly due to the inherent challenges of treating a population with significant cognitive and motivational impairments. The rise of technology presents an opportunity to develop novel treatments that may circumvent the motivational and cognitive challenges observed in schizophrenia.

**Objective:**

The purpose of this study was two-fold: (1) to evaluate the feasibility and acceptability of implementing a Personalized Real-Time Intervention for Motivation Enhancement (PRIME), a mobile app intervention designed to target reward-processing impairments, enhance motivation, and thereby improve quality of life in recent onset schizophrenia, and (2) evaluate the empirical benefits of using an iterative, user-centered design (UCD) process.

**Methods:**

We conducted two design workshops with 15 key stakeholders, followed by a series of in-depth interviews in collaboration with IDEO, a design and innovation firm. The UCD approach ultimately resulted in the first iteration of PRIME, which was evaluated by 10 RO participants. Results from the Stage 1 participants were then used to guide the next iteration that is currently being evaluated in an ongoing RCT. Participants in both phases were encouraged to use the app daily with a minimum frequency of 1/week over a 12-week period.

**Results:**

The UCD process resulted in the following feature set: (1) delivery of text message (short message service, SMS)-based motivational coaching from trained therapists, (2) individualized goal setting in prognostically important psychosocial domains, (3) social networking via direct peer-to-peer messaging, and (4) community “moments feed” to capture and reinforce rewarding experiences and goal achievements. Users preferred an experience that highlighted several of the principles of self-determination theory, including the desire for more control of their future (autonomy and competence) and an approach that helps them improve existing relationships (relatedness). IDEO, also recommended an approach that was casual, friendly, and nonstigmatizing, which is in line with the recovery model of psychosis. After 12-weeks of using PRIME, participants used the app, on average, every other day, were actively engaged with its various features each time they logged in and retention and satisfaction was high (20/20, 100% retention, high satisfaction ratings). The iterative design process lead to a 2- to 3-fold increase in engagement from Stage 1 to Stage 2 in almost each aspect of the platform.

**Conclusions:**

These results indicate that the neuroscience-informed mobile app, PRIME, is a feasible and acceptable intervention for young people with schizophrenia**.**

## Introduction

Schizophrenia is associated with significant psychosocial impairments, which lead to poor quality of life [1,2]. Recent data suggest that negative symptoms, and amotivation in particular, are the single most important factor affecting functional disability in schizophrenia [3,4]. Recent cognitive neuroscience research demonstrates that motivational deficits in schizophrenia are characterized by impairments in reward prediction, maintenance of rewarding experiences to guide future behavior, and a reduction in higher effort allocation to obtain rewarding experiences [5-8]. The likelihood to anticipate experiences to be less rewarding and more effortful leads to significant functional impairments. Yet, some aspects of reward processing for individuals with schizophrenia may be preserved, such the degree to which reward experiences are perceived as pleasurable in the moment. Findings suggest that individuals with schizophrenia demonstrate intact hedonic experiences, as evidenced by subjective reports of in-the-moment positive emotion [6,9,10], self-reported arousal similar to healthy comparison subjects [11], and intact hedonic experiences in social contexts [11]. While it appears that the ability to experience pleasure from rewarding experiences is intact, individuals with schizophrenia are more likely to be alone and endorse a preference to be alone while in the company of others, relative to healthy subjects [11], which suggests that the in-the-moment experience of pleasure may not be motivating future social interactions. Given the progress we have made in understanding the complexity of reward processing deficits in schizophrenia and how they might influence motivated behavior, it is time to translate these findings into enhancements of traditional treatments and/or developing novel approaches.

Current gold-standard treatments for individuals with schizophrenia include atypical antipsychotic medication and psychotherapy, with cognitive behavioral therapy (CBT) as one of the most effective behavioral treatments. These approaches are particularly effective at treating the positive, psychotic symptoms, but they do not adequately treat the negative symptoms [12,13]. While some of this may be due to problems with engagement and/or cognitive deficits, which limit the degree to which the skills may be used in real-world settings, the evidence suggests that the current gold-standard treatments do not lead to clinically meaningful improvements in motivated behavior, negative symptom severity, functioning, and quality of life [12,13]. Negative symptoms in schizophrenia, including social withdrawal, limited affective expression, and decreased drive to engage in motivated behavior, pose significant challenges to traditional treatment approaches that require patients to attend in person sessions and make use of the treatment in real-world contexts [14]. As such, it is not surprising that treatments that use compensatory strategies, such as environmental supports and reinforcement in simulated contexts (cues, reminders, and reinforcement), may be more effective in treating negative symptoms and improving psychosocial functioning, than traditional approaches [15-19]. What this suggests is that treatments that target negative symptoms, may benefit from using strategies that bypass cognitive impairments and deliver environmental supports in real-world contexts that directly motivate individuals with schizophrenia to engage in more rewarding experiences.

The rise of digital health technology presents an opportunity to develop novel treatments that may circumvent the motivational and cognitive challenges observed in schizophrenia. Further, a mobile approach delivered in real-time and in real-world settings, may support the retention, reinforcement, and transfer of intact in-the-moment hedonic experiences to future behavior. Mobile interventions may be accessed with greater frequency than traditional psychotherapy approaches and briefer therapeutic interactions might require less effort and have a greater therapeutic benefit. Indeed, several research groups are taking advantage of technology to deliver behavioral treatments to individuals with schizophrenia [20,21].

These considerations led us to harness digital technology and translate current knowledge on reward processing deficits in schizophrenia into an intervention to improve motivated behavior in the early phases of the illness. We thus designed and developed a Personalized Real-time Intervention for Motivational Enhancement (PRIME), a mobile app treatment that delivers text message (short message service, SMS)-based motivational coaching from trained therapists; individualized goal setting in prognostically important psychosocial domains; and social networking via direct peer-to-peer messaging as well as a community “moments feed” to capture and reinforce rewarding experiences and goal achievements. PRIME was designed with a systematic user-centered design process that included the involvement of individuals with schizophrenia, family members, treatment providers, and research experts. The purpose of this report is two-fold: (1) to evaluate the initial feasibility and acceptability of implementing the PRIME intervention to individuals who were recently diagnosed with schizophrenia, and (2) to evaluate the empirical benefits of using an iterative, user-centered design (UCD) process to develop a digital intervention for individuals with schizophrenia.

## Methods

### User-Centered Design Process

UCD, also referred to as human-centered design, is a process for gaining insight into the needs of end-users, creating novel approaches to meet those needs, and delivering solutions that are optimized for specific contexts [22,23]. Our team worked in collaboration with IDEO, a design and innovation firm, to develop our design strategy and implement the UCD process. Over a 4-week period, we conducted two design workshops with 15 key stakeholders (young individuals with a schizophrenia-spectrum disorder, family members, treatment providers, and research experts), and conducted a series of in-depth, 1:1 in-person interviews with six young people with schizophrenia-spectrum disorders.

The 1:1 interviews included several exercises focused on gaining a better understanding of the values that drive participants to improve their lives. During the initial design workshop, key stakeholders generated 12 potential values that would improve quality of life, including: (1) feel part of a group, (2) be a role model, (3) deepen my relationships with family and friends, (4) have a partner/boyfriend/girlfriend, (5) feel energized, (6) feel appreciated when I achieve a goal, (7) feel a sense of progress, (8) feel productive, (9) remember positive moments, (10) feel happy, (11) feel “normal”/not like I am ill, and (12) feel a sense of control over my future. During the 1:1 interviews, participants discussed whether those values were personally relevant and ranked them in order of importance. While most of the values were important to participants, the top two priorities for participants were to feel a sense of control over my future and deepen my relationships with family and friends. This is in line with self-determination theory (SDT) [24], which emphasizes relatedness, autonomy, and competence as essential values that drive intrinsically motivated behavior. The SDT framework has been used to understand motivated behavior in schizophrenia, such as supporting the motivating role of relatedness [25], the importance of autonomous choices in engaging in health-promoting behaviors [26], and competence in driving reward learning [25,27]. We therefore explicitly integrated principles of SDT into the overall design structure of the app.

Another objective of the interviews was to evaluate each feature of the app. For instance, we used experiential strategies such as prototyping specific features and presenting potential paper mockups of the app. An example of a prototyping exercise was to invite participants to use their mobile phones to take photos of positive moments in their life. Our team was interested in developing a feature that prompted participants to capture rewarding experiences and share those experiences among peers and coaches. When participants came in for their interviews, we reviewed their photos and evaluated the subjective experience of engaging in this type of activity. Notably, in addition to participants reporting that they enjoyed the process of taking the photos, they also became much more affectively expressive, talkative, and overall more engaged during this portion of the interview, suggesting that this feature would be well received and that participants enjoyed the opportunity to communicate nonverbally through pictures.

Lastly, we presented paper mockups of the app, which enabled our team to evaluate the design, flow, and functionality of particular features. An example of how this significantly influenced the final design of the app was observed during the evaluation of the goal-setting feature. Participants were shown two options of how a user might navigate the goal-setting feature. In one case, participants were shown a “daily challenge” option that was highly customizable and in the other case, the feature was prepopulated with personalized content and a simplified interface. While we expected that our participants would prefer a more customizable option, the degree of complexity and effort required to navigate a customizable experience was undesirable, as almost all the users preferred simplicity to customization. This subtle, yet highly important preference significantly influenced the design of this feature.

**Figure 1:**
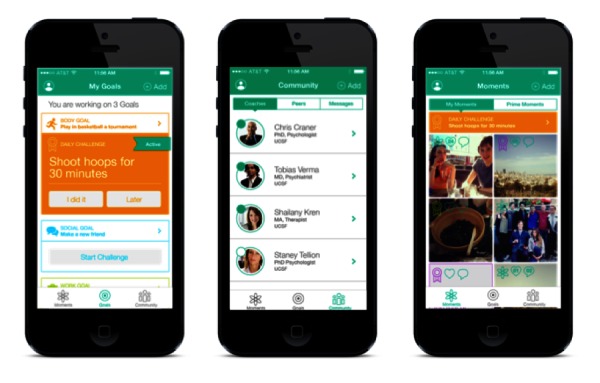
Screenshots of the three primary features of PRIME (from left to right): Goals (goal-setting), Community (Text-based motivational coaching), and Moments (social networking and community feed).

The UCD approach ultimately resulted in the first iteration of PRIME, a mobile app intervention aimed at improving motivation and functioning in people with recent-onset schizophrenia-spectrum disorders. With PRIME, participants join a supportive online environment where they can select and document progress on small, self-determined goals in four key domains that have been shown to be significantly associated with better quality of life: (1) health/wellness [28,29], (2) social relationships [30], (3) creativity [31], and (4) productivity [32]. PRIME provides users with motivation coaches: Masters-level clinicians who use evidence-based “microinterventions” drawn from CBT, mindfulness, and psychoeducation to help participants overcome the daily obstacles that hinder goal progress and PRIME engagement. Additionally, the PRIME community provides a platform for users to interact with one another. Users may send messages directly to each other and can also capture and share positive moments in their daily life with the community. Importantly, the PRIME app also includes a unique tone. Our participants noted during the design process that existing mental health apps and treatment approaches overly emphasize illness, and as such, IDEO suggested an approach that focused on a more casual, friendly, nonstigmatizing tone. Examples include the overall “look” of the app, which feels like a mainstream social media app, rather than a clinical tool, and the language used in the automated responses to completed challenges (ie, “You’re basically amazing. Way to rock that challenge!”). This approach is in line with the recovery model of psychosis, which emphasizes a collaborative, reinforcing and strengths-based approach [32,33]. The final design and screenshots of the PRIME app are shown in Figure 1.

### Procedures: Using an Iterative Design Process to Improve PRIME Feasibility and Acceptability

Importantly, and unlike what occurs in traditional psychotherapy randomized control trials (RCTs), the PRIME development and evaluation process was iterative and consisted of two stages. In Stage 1, our first iteration, we enrolled 10 participants to evaluate initial feasibility and acceptability. The results from the first 10 participants were then used in Stage 2 to inform the next iteration of PRIME to be tested in a RCT, during which participants were randomized to either receive PRIME or a wait-list/treatment as usual control condition. Participants in both stages were encouraged to use the app daily with a minimum frequency of 1/week over a 12-week period. The same assessment schedule was used for both stages and included clinical evaluations at baseline, 12-weeks, and a 3-month follow-up assessment. Outcome evaluators in the RCT are blind to condition. The RCT is ongoing and as such, we only report the feasibility results of the Stage 1 participants and the first 10 randomized participants from Stage 2. Participants were compensated for their time to complete study-related assessments (US$20/hour), but were not paid for their participation in the intervention.

### PRIME Protocol

Once participants completed their baseline assessments, participants were either provided with an Apple iPhone 5C with PRIME already installed or a study coordinator installed PRIME on their own personal iPhone. Participants initially met with an assigned “motivation coach” for a “set-up session.” The purpose of the set up session is to orient participants to the app and to discuss goals the participant would like to achieve by using PRIME. The first time participants sign in to the app, they are guided through a process of creating a user profile. Participants create a user name, upload a profile picture, select their interests, goals, symptoms, and write a short bio. Goals and interests are categorized in the domains of health/wellness, social, productivity, and creativity. Participants are given the option to make their profile “private” or “public” to the PRIME community. If users select private, their peers will not be able to see their profile or posts to the community feed. However, coaches will be able to view their profile and posts in a Web-based “backoffice.” Participants are informed of the privacy practices in the app and encouraged to be respectful of their peers’ privacy.

Once the user is set up with his/her profile, the participant may select from prepopulated, personally tailored “daily challenges,” associated with a participant’s stated goals. For instance, if a participant endorsed “working out” as an interest and a goal of “feeling healthier,” the participant will be shown a daily challenge of “Spend X number of minutes working out today.” The participant may select the amount of time spent engaging in that daily challenge and set an alert to remind him/her to work on that particular daily challenge. Once a participant has completed their challenge, they have the option to post the goal achievement to the community or to their own private feed (also viewable to coaches in the backoffice). Participants are also encouraged to post spontaneous, positive moments in their life to encourage reinforcement of rewarding experiences. The daily challenge and spontaneous moments are displayed on the PRIME community feed and the participants’ “My PRIME” feed. Assigned coaches explain their role and immediately engage the user in a text-based chat about the participant’s goals. For the Stage 1 participants, the coaches explained to their participants that they would reach out to them 1/week, but that the participants could message them at any time they would like help working on a goal or anything that might interfere with goal achievement. Based on use patterns and feedback from participants (reported below) the protocol was changed and coaches now explain that they will reach out to the user on “most days” (on average 4 days per week), but will modify the frequency depending on the user’s preference, clinical issues, and degree of overall progress toward goal achievement. Participants are also shown the option to view their peers’ profiles and shown how to message with them directly. Other opportunities for social interaction include “liking” or commenting on moments posted to the PRIME community feed.

#### PRIME Coach Training

The coaching staff currently consists of six Master’s level clinicians who are experienced delivering CBT to patients in community practice settings. Coaches are trained over a 2-hour session using the PRIME treatment manual, which orients coaches to the clinical population, evidence-based approaches to managing symptoms, including CBT, psychoeducation, and mindfulness techniques, and suggested tone/approach to match the overall tone of the app. Due to the text-based modality of coaching, the manual discusses suggested adaptations, such as emphasizing more behavioral strategies during asynchronous interactions, delivering Web-based resources, such as short videos or websites providing psychoeducation about schizophrenia or other distressing symptoms, cognitive strategies, activity planning, and other behavioral suggestions. After an initial training, coaches “round” on their participants during a weekly meeting with the principal investigator and coaching team to review cases and troubleshoot potential challenges.

### Participants

A total of twenty people (10 in the Stage 1 group and 10 in the Stage 2 group) met Diagnostic and Statistical Manual-IV-TR (American Psychiatric Association, 2000) criteria for a schizophrenia spectrum disorder: schizophrenia (n=13), schizophreniform (n=2), or schizoaffective disorder (n=5). Participants were recruited from the Early Psychosis Clinic at University of California, San Francisco as well as other community-based treatment providers in the San Francisco Bay Area. In addition to local recruitment, several participants (n=3) living outside of California were remotely enrolled from Texas, Maryland, and North Carolina. Participants were between the ages of 16 and 30 and in their early course of illness, defined as being within the first 5 years of formal diagnosis. In addition, participants had no history of neurological disorders or serious head trauma, were fluent in English, had an estimated intelligence quotient (IQ) > 70, and did not meet criteria for a substance dependence disorder within the past 6 months. Demographic information, such as age, years of education, as well as use of treatment resources (eg, therapy, psychiatric services) can be found in Table 1.

**Table 1:** Demographic and clinical characteristics.

		Stage 1	Stage 2	
		(n=10)	(n=10)	*t* or *x* ^2^ (*P)*
		Mean (SD)	Mean (SD)	
Age (years)		23.40 (2.6)	23.30 (3.7)	.95
n, (%) Male		8/10 (80%)	9/10 (90%)	.63
Education (years)		14.40 (1.6)	14.00 (1.9)	.62
Duration of Illness (months)		46.40 (20.3)	27.80 (16.5)	.04
Racial Background, n (%)				
	Caucasian	3 (30%)	3 (30%)	.61
	Asian	4 (40%)	2 (20%)	
	African American	2 (20%)	2 (20%)	
	Other	1 (10%)	3 (30%)	
n, % seeing therapist		6 (60%)	6 (60%)	.83
n, % seeing psychiatrist		4 (40%)	8 (80%)	.06
WTAR^a^ FSIQ		106.90 (8.7)	108.90 (6.6)	.57
PANSS^b^				
	Positive total	6.00 (2.8)	6.70 (4.3)	.75
	Negative total	11.60 (6.6)	12.80 (6.6)	.67
	Overall total	53.60 (12.9)	55.60 (12.2)	.69
RFS^c^				
	Work Productivity	5.50 (1.5)	4.30 (1.7)	.11
	Independent Living	6.00 (1.2)	5.20 (0.9)	.10
	Social Networks	5.30 (1.9)	4.70 (1.6)	.46
	Family	6.30 (.94)	6.250 (.53)	.57
QOL-A^d^		38.60 (11.2)	33.10 (9.1)	.25

^a^Wechsler Test of Adult Reading.

^b^Positive and Negative Syndrome Scale.

^c^Role Functioning Scale.

^d^Quality of Life Scale-Abbreviated.

### Clinical and Interview-Based Assessment

Trained interviewers confirmed diagnoses using the Structured Clinical Interview for the Diagnostic and Statistical Manual-IV [34]. We assessed positive and negative symptoms using the positive and negative syndrome scale (PANSS) [35]. Functioning in the areas of work, self-care, family, and social was assessed with the role functioning scale (RFS) [36]. Quality of Life was assessed with the quality of life scale - abbreviated (QOL-A) [37]. See Table 1 for symptom, functioning, and quality of life characteristics. We estimated full-scale IQ with the Wechsler Test of Adult Reading (WTAR). For remote participants, the clinical and interview-based assessment was conducted via FaceTime or Skype.

### Assessing PRIME Acceptability

We assessed PRIME acceptability during an exit interview where participants rated their satisfaction with the specific features of PRIME, such as the ability to interact with peers and the different goal categories, on a 1 (not at all) to 10 (very much) scale. We also assessed retention in the trial as a measure of acceptability.

### Assessing PRIME Feasibility

To evaluate feasibility, we assessed the following: login frequency (average number of days logging in per week), average number of challenges completed (both overall and by individual challenge category), challenge completion percentage, and the average number of peer and coach interactions. Interactions included direct messaging on PRIME as well as commenting on and liking content posted to the community moments feed. To further understand how participants were engaging with the PRIME platform, we evaluated user metrics, such as active use (ie, while logged in, any type of interaction with coaches and/or peers, posting spontaneous or goal achievement moments) versus passive use (logging in, but not engaging with peers or coaches or posting content onto the moments feed), the degree of social reciprocity (ie, initiating interactions and the ratio of initiated interactions to responsive interactions) from peer-to-peer and coach-to-peer interactions.

### Data Analysis Plan

We examined whether any demographic variables were related to symptoms or functioning in the overall sample, and whether any demographic variables, symptoms, or functioning were related to PRIME use by conducting zero-order correlations. We also investigated any group differences (Stage 1 vs Stage 2) in demographic variables, symptoms, and functioning by conducting independent samples *t*-tests for continuous variables and chi-square tests for categorical variables. To examine initial acceptability of PRIME, we compared the average ratings for each group from the PRIME exit interview for overall satisfaction as well as the most and least popular PRIME features using independent samples *t*-tests. Furthermore, we also reviewed qualitative feedback from the PRIME exit interview.

To investigate initial feasibility of PRIME, we examined descriptive statistics for the following PRIME metrics: login frequency, challenges completed, peer and coach interactions, and active use rate. To understand participants use of PRIME on a daily basis, we computed a variable that represented how often participant’s actively used PRIME, which we called the active use rate. To do this, we added together the average number of challenges completed, peer, and coach interactions and divided this total by the number of weeks the participant had access to PRIME. Thus, a value of 2.3 would mean that a participant was active on PRIME 2.3 times/week. Passive use was defined as logging into the app, but not posting a moment, completing a challenge or interacting with peers or coaches. Thus, a participant may login to the app 4 days/week, but actively engage with the features of the app 2 days/week.

In addition to examining the total number of coach and peer interactions, we also calculated the degree to which participants initiated interactions, relative to received interactions as an indicator of engagement. For instance, a ratio of 3:1 for peer-to-peer interactions would mean that for every three times a user sent a message, the peer received one message, which suggests that the user is more proactively engaging in interactions. For both PRIME acceptability and feasibility, we tested the effectiveness of our iterative design process by comparing the Stage 1 and Stage 2 participants using independent samples *t*-tests, correcting for multiple comparisons.

## Results

Participant demographics are reported in Table 1. Participants in the Stage 1 group had a significantly longer duration of illness than the Stage 2 group. However, neither this nor any other demographic variable was related to symptoms or functioning in either group, nor were symptoms and functioning related to PRIME use in the two groups.

We also assessed participant digital health use (eg, health-related mobile app usage) over the past month. In terms of digital health resource usage, 75% (15/20) reported owning a smartphone. We then asked participants whether they used digital health apps in their daily lives. Overall, participants reported low levels of digital health use, with the most used applications falling in the categories of increasing exercise and fitness (5/20, 25%), improving relaxation (3/20, 15%), and improving mood (2/20, 10%). Only one of the 20 participants reported using digital health resources for managing weight or alcohol use. In comparison, 95% (19/20), reported using a social media platform (e.g. Facebook, Twitter), with 50% (10/20) also using social media for sharing photos and music. Participants in Stage 1 reported using social media apps daily and participants in Stage 2 reported social media use “more days than not.”

### PRIME Acceptability

To date, all of the participants were retained in the trial. Mean overall satisfaction with PRIME for the entire sample, as rated during the exit interview administered at the 12-week post-assessment, was 8.00 (standard deviation (SD): 2.0). The difference between the two groups was not significant (Stage 1: mean: 7.25, SD: 2.3; Stage 2: mean: 8.86, SD: 1.2). Some of the comments made by participants when asked about how PRIME influenced their lives included:

There’s nothing like it out there

It was a good chance to be more social, meet other people, and see what they're like.

Definitely getting to do more, not chores, but be more active. Motivation to do more stuff, achieve more goals, and be more social.

The most popular PRIME features for both groups were the ability to comment on other user’s posts (mean: 8.53, SD: 1.9) and the least popular PRIME feature was the ability to view coach profiles (mean: 7.33, SD: 2.7).

### PRIME Feasibility

PRIME use data (login frequency, challenge completion, and interactions) for the participants in both stages are shown in Table 2. Overall, participants logged into PRIME approximately every other day, with the Stage 2 sample logging in at a slightly higher frequency. Over a 12-week period, participants were highly engaged in the platform, with 177 direct messages sent from participants to coaches in the Stage 1 sample and 955 sent from participants to coaches in the Stage 2 sample (*P*=.04; *d*=1.02). In terms of peer-to-peer interactions, participants initiated interactions with each other 97 times in the Stage 1 sample and 151 times in the Stage 2 sample (*P*=.49, *d*=.23).

**Table 2:** PRIME usage data for the overall sample as well as both the Stage 1 and Stage 2 groups.

		Stage 1	Stage 2	
		Mean (SD)	Mean (SD)	*p, d* [95% CI]
Login frequency (average logins per week)		3.51 (1.8)	4.69 (1.4)	.13, .73 [-.20. to 1.60]
Challenge completion rate (%)		84.48 (18.4)	85.35 (15.3)	.91, .05 [-.83 to .93]
Average number of user-initiated peer interactions				
	Comments	7.70 (6.4)	9.50 (10.1)	.68, .21 [-.67 to 1.08]
	Likes	11.80 (11.9)	24.00 (20.6)	.12, .73 [-.21 to 1.60]
	Messages	9.70 (8.8)	15.10 (22.5)	.49, .32 [-.58 to 1.18]
	Total	29.20 (21.1)	48.60 (44.3)	.23, .56 [-.36 to 1.43]
Average number of user-initiated coach interactions				
	Comments	7.80 (7.0)	12.90 (14.5)	.33, .45 [-.46 to 1.32]
	Likes	4.40 (4.9)	10.00 (12.3)	.20, .60 [-.06 to 1.47]
	Messages	17.70 (22.9)	95.50 (105.8)	**.04** ^a^, 1.02 [.05 to 1.90]
	Total	29.90 (31.6)	118.40 (105.9)	**.02**, 1.13 [.15 to 2.02]
Challenges completed				
	Overall	18.40 (14.5)	20.40 (18.2)	.79, .12 [-.76 to .99]
	Health/wellness	6.80 (5.7)	7.80 (9.1)	.77, .10 [-1.47 to .49]
	Social	3.20 (1.8)	4.40 (3.9)	.39, .40 [-.51 to 1.26]
	Creativity	4.50 (4.7)	4.40 (3.7)	.96, -.02 [-.85 to .90]
	Productivity	3.90 (5.7)	3.80 (3.6)	.95, -.02 [-.86 to .90]
Active engagement rate (average times active per week)		3.87 (2.7)	11.17 (9.4)	**.03**, 1.06 [.08 to 1.94]

^a^Bold Values indicate statistically significant results.

Participants in both groups completed an average of approximately 1.5 challenges per week, with the Stage 1 sample completing slightly more than the Stage 2 sample. For both groups, health/wellness challenges were the most popular at approximately one challenge completed per week, followed by creativity challenges, social challenges, and productivity challenges of which participants completed approximately 1 every 2 weeks. Challenge completion percentage was high for both groups (>16/20, 80%), suggesting that participants had little difficulty completing the challenges that they set. Participants had approximately two interactions with coaches and two interactions with peers each week. Participants in the Stage 2 sample tended to have more interactions with coaches and peers than those in the pilot group. The Stage 1 group, on average, was active on PRIME approximately 4 times/week. This rate almost tripled for the Stage 2 group at a little over 11 times/week (see Table 2). The degree to which participants reciprocated interactions initiated by a peer or coach user is shown in Table 3. The ratios reflect a higher degree of reciprocity in peer interactions than coach interactions. Yet, the Stage 2 participants were much more responsive to coach interactions than the Stage 1 participants (see below for a description of the change in the coaching strategy). Real examples of peer to peer and coach to peer interactions are shown in Textboxes 1 and .

**Examples of peer to peer interactions.**

John: Hey the doctors said I should connect with you because you have some good insights about the diagnosis. What do you think about it?

David: Hey bud, sorry about the delayed response. Thanks for reaching out. I think this disease is definitely a huge challenge that we all sort of have to face. But I think its a type of challenge that can be overcome. When I first got the diagnosis, I was a wreck. Depression as well as an onslaught of negative symptoms... I think it's important to slowly get ur life back together step by step.  As u progress, u will realize things really are not all that bad and that this disease is just one of the bumps in life that u will have to overcome.

John: I agree, it's just another challenge. How do you deal with your negative symptoms?

David: It takes time. Therapy helped.  Also a goal orientated attitude is important. Gotta realize that u r no different than someone without the disorder after proper medical treatment.

**Examples of and coach to peer interactions.**

Coach: Hey [participant’s name]! I noticed you haven’t completed any challenges or posted any moments recently. What have you been up to?

Participant: Playing video games. I haven’t been up to it lately. Is there a way I can fight through this? Laziness or whatever it’s called? How do you complete your goals in life? I feel stagnant more depressed and less motivated. I feel like this because I have low self esteem. So I play video games to distract myself and get lost. Doing much seems unrealistic.

Coach: I’m sorry to hear that you haven’t been feeling great recently. Feeling blue happens to us all from time to time, and luckily there are some ways to combat feeling this way. One thing that might help is trying to be active. I know it’s hard when you feel this way, but even something as simple as going for a walk can help. When you feel this way, do you notice that you are having negative thoughts as well?

Participant: Yeah ok Coach thank you. I love being active, working out, cleaning, walking my dogs, doing hw, cooking. These things help me focus.

Coach: Do you notice feeling different? Sometimes it helps to take a strep back and realize all that you have accomplished. You have worked very hard over the past 2 months and have made good progress. I am proud of you and hope that you feel proud as well.

Participant: Thank you so much you are right I have!!! I feel like I’m not going to be so harsh on my self and I feel like being more active!

Coach: Self-compassion is important as being too hard on yourself can make you feel down. Next time you think you are being hard on yourself, try writing down some things you recently accomplished. Or, you can write down evidence for and against the thoughts you are having. I have used both approaches and they both work!

Participant: Okay thank you so much. I’m going to try and write down some recent accomplishments and find evidence against being too hard on myself.

Coach: Great to hear! Let me know how it works. Maybe you can complete a challenge this weekend.

### Iterative Design Process Results: Shifting the Coaching Strategy and Tone

The feasibility and acceptability results from the Stage 1 participants, reported above, for the most part demonstrated that the intervention was well tolerated, yet feedback from individual participants coupled with an examination of their use patterns suggested we should modify our approach to motivation coaching. For instance, one participant felt that it seemed that the coaches were working “off of a script,” which made it hard for her to connect with her coach, stating:

It felt like every time [my coach] posted something, she was fitting me inside this box. Instead of having a normal conversation like "Hey what's up, how are midterms", they followed a script.

Still, others found the coaching to be very helpful, with one participant stating:

It was great! I think the coaches were very, very, very supportive and they did as much as they could to help me participate and be active in the community. They helped me manage my stress and take on everyday life and they did a good job.

Another key observation during the first iteration was the low response rate to coach initiated interactions (Table 3), with less than half of the messages sent by coaches receiving a response from participants. The ratio of coach to participant responses was 12:1 for the pilot participants. This led to the following changes in Stage 2: (1) we increased the frequency of the interactions initiated by motivation coaches from once a week to an initial 5 days/week followed by a decreased frequency over time, based on the preference of the participant and their clinical needs, (2) decreased the amount of content in each message, and (3) personalized the messages and adopted a more casual tone that reflected the tone recommendations by IDEO. For example, instead of sending a lengthy, once per week message, coaches would instead send a brief message most days per week, checking in with their participant (eg, “Hi there! I’d love to see you work on your goal to exercise more. How about going for a walk today?”). By initiating almost daily contact, we sought to increase overall engagement with PRIME and to facilitate more frequent interactions with coaches and among peers. By decreasing the amount of content in each message, we aimed to reduce the cognitive load for participants and make it easier to respond to messages. The data suggest that this change may have led to increased engagement as evidenced by a 2- to 3-fold increase in the following usage patterns between Stage 1 and Stage 2: number of logins (3.51 days/week to 4.69 days/week), overall peer and coach interactions (peers: from 29.20-48.60 (*P*=.23, *d*=.56); coaches: from 29.90-118.40 (*P*=.02; *d*=1.13)), active use rate (~4 times/week to ~11 times/week; see Table 2), and the degree of coaching interaction reciprocity (Table 3).

**Table 3:** Initiated to received coaching and peer interaction rate for Stage 1 and Stage 2 groups.

		Stage 1	Stage 2	*P, d* [95% CI]
		Mean	Mean	
Coach interaction (ratio of interactions initiated by coaches to received)				
	Comments	10.88**:**1	8.16**:**1	44, -.37 [-1.24 to.53]
	Like	44.40**:**1	24.17**:**1	.19, .-.65 [-1.52 to .27]
	Messages	5.17**:**1	1.87**:**1	.07, -.87 [-1.75 to .08]
	Overall	12.23**:**1	3.37**:**1	.01^a^, -1.35 [-2.26 to -.33]
Peer interaction (ratio of interactions initiated by peers to received)				
	Comments	1.68 **:** 1	1.89 **:** 1	.81, .12 [-.77 to .99]
	Like	2.06 **:** 1	0.98 **:** 1	.42, -.41 [-1.28 to .49]
	Messages	1.10 **:** 1	1.03 **:** 1	.65, -.20 [-1.07 to .69]
	Overall	1.17 **:** 1	1.25 **:** 1	.84, .10 [-.78 to .97]

^a^Statistically significant result.

## Discussion

### Principal Findings

The results from this study demonstrated that the cognitive neuroscience-informed mobile app, PRIME, is a feasible and acceptable intervention for young people with schizophrenia**.** Over 70% (14/20) of our participants reported owning a smartphone and 95% (19/20) reported using social media, suggesting that using a smartphone-based, social networking platform to deliver an intervention would be an acceptable treatment modality for this population. The overall satisfaction with PRIME was relatively high, as reflected in the satisfaction ratings endorsed in the exit interview as well as the current 100% (20/20) study retention rate. The PRIME use data demonstrated a high degree of engagement with this digital treatment platform. Participants used the app, on average, every other day, and were actively engaged with its various features each time they logged in. Qualitative feedback from participants was overall positive, and the critiques of the platform during Stage 1 were used to guide refinements for Stage 2 (ie, the coaching strategy). A common theme in the feedback from participants was the positive experience of social support from coaches and their peers. This was also reflected in the total number and frequency of interactions and degree of reciprocity the participants exhibited with their coaches and peers. The quality of the interactions, as reflected in the provided examples, was also impressive given the entirely text-based messaging format.

The increase in various metrics of engagement between Stage 1 and Stage 2 participants, suggests that the integration of user feedback to influence the refinement of the intervention was successful. The changes we made in response to this user-centered, iterative approach led to a 2- to 3-fold increase from Stage 1 to Stage 2 in use of the app (ie, logins), greater active use (engagement with features), a significantly greater number of social interactions with peers and coaches and improved ratios of reciprocal interactions with coaching. While the changes we made were focused on the text-based coaching, these simple changes appeared to generalize to greater engagement in almost every aspect of the platform. Taken together, this suggests that the development of mobile digital interventions should continuously incorporate user feedback and adopt refinements to meet the needs of the population. This is in line with conclusions from the Agency for Healthcare Quality Improvement, which recently highlighted that the success of new technology-based interventions hinges on incorporating user feedback in design and implementation [38].

### Limitations

This study has several limitations. First, our sample size is relatively small, which limits the generalizability of the results. However, the tradeoff for using a small sample size in Stage 1 was that we were able to very quickly evaluate the feasibility of the app and refine the platform for use in Stage 2. Another limitation of this feasibility study is that our participants were all in the early phase of schizophrenia and therefore the results of the study are likely not representative of the larger population of those with persistent schizophrenia. We decided to focus on designing the intervention to treat young patients early in their course of illness based on numerous findings promoting the benefits of early intervention [39,40]. By designing an intervention specifically for use with this population we are aiming to significantly improve the course of illness by improving functional outcome during a critical period.

### Conclusions

With the proliferation of digital technology-based interventions to address mental health issues, it is encouraging to learn that a mobile app-based treatment is feasible, tolerable, and acceptable to young people with schizophrenia. Indeed, several other research groups have developed novel digital technologies to improve outcome for individuals with schizophrenia, often emphasizing CBT approaches for positive symptoms and focusing on people with persistent illness [29,41]. In addition, the existing digital treatment tools have not been developed with an explicit cognitive neuroscience rationale, have not focused on the needs of recent onset individuals, and have not included a social network with peers or mental health coaches [21]. The results of our feasibility study seem to suggest that the delivery of a smartphone mobile app intervention that includes several opportunities for social engagement and to share goal achievements within a recovery-oriented framework is a desired treatment modality for young people with schizophrenia. The goal of our ongoing RCT will be to explicitly examine the degree to which the focus on targeting reward processing impairments and enhancing motivated behavior will lead to improved functional outcome in this population.

## Abbreviations

CBT: cognitive behavioral therapy
IQ: intelligence quotient
PANSS: positive and negative syndrome scale
PRIME: personalized real-time intervention for motivation enhancement
QOL-A: quality of life scale – abbreviated
RCT: randomized control trials
RFS: role functioning scale
SD: standard deviation
SDT: self-determination theory
SMS: short message service
UCD: user-centered design
WTAR: Wechsler test of adult reading
